# Dilemma between health and environmental motives when purchasing animal food products: sociodemographic and nutritional characteristics of consumers

**DOI:** 10.1186/s12889-017-4875-6

**Published:** 2017-11-10

**Authors:** Sandrine Péneau, Philippine Fassier, Benjamin Allès, Emmanuelle Kesse-Guyot, Serge Hercberg, Caroline Méjean

**Affiliations:** 1 Université Paris 13, Sorbonne Paris Cité, Equipe de Recherche en Epidémiologie Nutritionnelle (EREN), Centre de Recherche en Epidémiologies et Biostatistiques, Inserm (U1153), Inra, Cnam, COMUE Sorbonne Paris Cité, F-93017 Bobigny, France; 2 Université Paris 13, Sorbonne Paris Cité, Equipe de surveillance et d’épidémiologie nutritionnelle (ESEN), Santé Publique France, F-93017 Bobigny, France; 3 Département de Santé Publique, Hôpital Avicenne, F-93000 Bobigny, France; 4 INRA, UMR 1110 MOISA, F-34000 Montpellier, France; 5 EREN, CRNH Ile-de-France, UFR SMBH Paris 13, Sorbonne Paris Cité, 74 rue Marcel Cachin Cedex, 93017 Bobigny, France

**Keywords:** Food motives, Sustainability, Dilemma, Health, Environment, Epidemiology

## Abstract

**Background:**

Dietary guidelines in France give quantitative recommendations for intake of meat, fish and dairy products whereas consumers are increasingly concerned by the environmental impacts associated with the production of these foods. This potentially leads to consumer dilemmas when purchasing food products. The present study aimed at investigating the sociodemographic profiles of individuals reporting health and environmental dilemmas when purchasing meat, fish and dairy products, and comparing diet quality of individuals with and without dilemma.

**Methods:**

A total of 22,936 adult participants in the NutriNet-Santé cohort were included in this cross-sectional analysis. Participants completed a questionnaire assessing motives when purchasing meat, fish and dairy products, including health and environmental determinants. Environmental vs. health dilemmas were assessed using implicit and explicit methods. Sociodemographic data as well as dietary intake using repeated 24 h–records were collected. The association between sociodemographic characteristics and presence of dilemma was assessed using logistic regression models and between dilemma and intake of these products, adherence to food group guidelines, or overall dietary quality, using covariance analysis.

**Results:**

Among participants, 13% were torn between buying meat for health reasons and to avoid buying it for environmental reasons, 12% in the case of fish and 5% in the case of dairy products. Older participants, women and low income individuals were more likely to report dilemmas. Participants reporting dilemmas for meat and dairy products consumed less of these foods (*P* < 0.05 and *P* < 0.0001, respectively) and had a better dietary quality overall (both *P* < 0.0001). In addition, participants with meat dilemma showed a better adherence to meat/fish/eggs guidelines (*P* < 0.001).

**Conclusions:**

Individuals reporting dilemmas concerning animal products had specific sociodemographic characteristics and showed higher diet quality overall compared with those having no dilemma. Our data suggest that having environmental concerns is not contradictory with adherence to nutritional guidelines.

## Background

Nutrition is one of the most important risk factors associated with development of chronic diseases, such as cardiovascular disease, some types of cancers, and type 2 diabetes [1, 2]. During the past decades, many countries in Europe and North America have set up nutritional policies to prevent or decrease these public health burdens [3, 4]. In France, a nutritional policy (PNNS) was launched in 2001 and dietary guidelines have been widely disseminated within the population in order to attain public health objectives [5]. In 2008, individuals aged 12–75 years were largely aware of these guidelines and in particular around two third were aware of the recommendation for “meat, fish and seafood, eggs” (once or twice a day), three-quarter were aware of the recommendation for “fish and seafood” specifically (at least twice a week) while around one fourth were aware of the recommendation for “dairy products” (three to four times a day depending on age) [5]. Subjects aware of guidelines for dairy products were two times more likely to achieve corresponding dietary recommendations, while the association was even stronger for fish [6].

Food production has been shown to account for 30% of greenhouse-gas emissions, thus contributing to climate change [7]. Consequently, it has been suggested to reduce both the intensity of emissions from livestock production and the average consumption level of animal products [8]. A large number of individuals are now concerned by these environmental issues associated with food choice [9–11] but translation into actual sustainable food choice and consumption seems difficult [12–15]. Underlying reasons include level of involvement with sustainability, perceived consumer effectiveness, perceived availability of sustainable products or social norm for example [15]. Other reasons might involve differences between guidelines focusing on health and environmental issues [16]. It is now recognized that climate change and chronic diseases must be tackled together to ensure coherent dietary advice for consumers [17, 18]. However, only a few countries have developed guidelines for their citizens that integrate health and environment when making food choices [19]. In countries where this is not the case, dilemmas between health and environment when making purchasing decisions are likely to occur. However, to our knowledge this has not been investigated in the literature. In England, it has been shown that about one-fifth of the subjects were confused about which type of fish to eat for health reasons, while it was the case for half of them when attempting to protect fish stocks [20]. This issue is likely to be even more significant for consumers who want to take into account both health and environmental aspects when making purchasing decisions.

Therefore, the present study aimed at investigating the existence of dilemmas between health and environmental motives when purchasing meat, fish and dairy products, at determining the sociodemographic profiles of individuals reporting dilemmas, and finally at comparing dietary quality of these individuals with those reporting no dilemma.

## Methods

### Study population

The NutriNet-Santé Study (https://www.etude-nutrinet-sante.fr) is an ongoing web-based prospective observational cohort study launched in France in May 2009 with a scheduled follow-up of 10 years. It aims to investigate the relationship between nutrition and chronic disease risk, as well as the determinants of dietary behavior and nutritional status. The study was implemented in the general French population (internet-using adult volunteers, age ≥ 18 years). The rationale, design and methodology of the study have been fully described elsewhere [21]. In brief, volunteers are recruited via multimedia campaigns (television, radio, national and regional newspapers, posters, internet) and invited to visit the NutriNet-Santé website. In order to be considered included in the study, participants complete a baseline set of self-administered, web-based questionnaires assessing dietary intake, physical activity, anthropometric characteristics, lifestyle, socioeconomic conditions and health status. As part of the follow-up, participants are requested to complete the same set of questionnaires every year. Moreover, each month, participants are invited by e-mail to fill in optional questionnaires related to dietary intakes, determinants of eating behaviors, nutritional and health status. This study is conducted in accordance with the Declaration of Helsinki, and all procedures were approved by the Institutional Review Board of the French 75 Institute for Health and Medical Research (IRB Inserm n° 0000388FWA00005831) and the *Commission Nationale de l’Informatique et des Libertés* (CNIL n° 908,450 and n° 909,216). All participants provided informed consent with an electronic signature. This study was registered in EudraCT on 25th February 2013 (n°2013–000929-31).

### Questionnaire measuring dilemmas for food choice motives during purchasing

Food choice motives during purchasing in general and for specific food groups (meat, fish, fruits and vegetables, dairy products) were assessed in September 2013 using an optional web-based questionnaire which was internally validated using factor analyses [11]. The present analyses focused on the following food groups: meat, fish and dairy products. In addition, dilemma for choice of meat, fish and dairy products was assessed with two approaches.

#### Implicit assessment of dilemma

The following questions of the questionnaire have been used for the present analyses: (i) “I purchase [meat/fish/dairy products] for health issues” (concerned participants who reported to purchase within the food group only), (ii) “I avoid purchasing [meat/fish/dairy products] for environmental issues” (concerned both participants who reported to purchase within the food group and those who reported no purchase). The subjects were asked to rate each item on the following 4-point Likert scale: strongly disagree/disagree/agree/strongly agree. If they could not answer the question, participants were allowed to choose the answer « I do not know ». Participants were defined as having a dilemma towards food purchase when they “agree” or “strongly agree” with both statements (i) and (ii).

#### Explicit assessment of dilemma

Additional questions were asked to the participants: (iii) I am torn between “purchasing [meat/fish/dairy products] to follow dietary guidelines” or “limit purchase for environmental issues”. The subjects were asked to answer each item on a yes/no scale. French dietary guidelines were mentioned for each food group. For meat: “eat meat, fish, eggs 1 to 2 times/day” since there is no guideline specific to meat; for fish: “eat fish twice/week”; and for dairy products: “eat dairy products (milk, yoghurt, cheese etc.) 3 to 4 times/day”. Participants were defined as having a dilemma towards food purchase when they answered yes to the question (iii).

#### Composite variable

Participants were defined as having a dilemma toward choice of meat, of fish or of dairy products if they were qualified as having a dilemma using both implicit and explicit approaches.

### Sociodemographic, economic and lifestyle data

The sociodemographic, economic and behavioral data that were the closest to the food choice motives questionnaire were used. In particular, data were collected on sex, age (years), BMI (kg/m^2^), educational level (primary, secondary, university (≤ 3 years), university (> 3 years)), household composition (one adult, two adults, household with at least one child, household with at least one teenager, household with at least one child and one teenager), smoking status (never smoker, former smoker, current smoker) physical activity (low, medium, high) and monthly household income (<1200, 1200–1800, 1800–2700, >2700 euros per household unit). The monthly household income is calculated per household consumer unit (CU). One CU is attributed to the first adult in the household, 0.5 CU - for other persons aged 14 or older, and 0.3 CU - for children under 14. Physical activity was assessed using a short form of the French version of the International Physical Activity questionnaire (IPAQ) [22]. The weekly energy expenditure expressed in metabolic equivalent task minutes per week was estimated, and 3 scores of physical activity were constituted [ie, low (<30 min/d), moderate (30–59 min/d), and high (≥60 min/d)] according to the French guidelines for recommended levels of physical activity [5].

### Food group intake, adherence to food group guidelines, and dietary quality (mPNNS-GS score)

At inclusion and once a year thereafter, participants are invited to complete three non-consecutive 24-h dietary records, randomly assigned over a 2-week period (2 week days and 1 weekend day). The accuracy of web-based 24 h dietary records has been assessed by comparing to interviews by trained dietitians [23] and against 24 h urinary biomarkers [24, 25]. For the present analysis, participants who had completed at least three 24 h dietary records during the two years preceding the questionnaire on food choice motives were selected. Participants reported all foods and beverages consumed at each eating occasion. They estimated the amounts eaten using validated photographs of portion sizes [26] using household measures or by indicating the exact quantity (g) or volume (mL). For each participant, daily mean intakes were calculated from the 24-h dietary records, weighted for the type of day of the week. Nutrient intakes were estimated using the published NutriNet-Santé composition table including more than 2000 foods [27]. Dietary underreporting was identified on the basis of the method proposed by Black [28].

For the purpose of this study, analyses focused on intake (g/100 g) of meat (red meat, poultry and game, organ meat), fish (fish, shellfish) and dairy product (milk, cheese, yoghurt) groups. The level of adherence to French dietary guidelines for fish (≥ 2 servings/week), dairy products (intake between 2.5–3.5 servings/day (individuals <55 years) and between 2.5–4.5 servings/day (individuals ≥55 years) was also assessed [5]. Since there are no specific dietary guidelines available for meat, guidelines for meat/fish/eggs were considered (≥1–2 servings/day) [5]. Finally, the mPNNS-GS was calculated. The mPNNS-GS is an a priori score assessing overall adherence to the French nutritional recommendations, and therefore reflecting overall dietary quality. It is based on the PNNS-GS score [29] but accounts for dietary component only, excluding the physical activity component [30]. Briefly, the score has a range of 0–13.5 points, with a higher score indicating a better overall diet quality. It includes 12 components: eight refer to food-serving recommendations (fruit and vegetables; starchy foods; whole grain products; dairy products; meat, eggs and fish; seafood; vegetable fat; water vs soda) and four refer to moderation (added fat; salt; sweets; alcohol). Negative points apply for overconsumption of salt (>12 g/day), added sugars (>15% energy intake). A penalty also applies when energy intake exceeds the energy requirement – as assessed by physical activity level and basal metabolic rate calculated using Schofield eqs. [31] – by more than 5%.

### Statistical analysis

Characteristics of included and excluded participants were compared using Student’s t tests or chi-square test, as appropriate. The degree of agreement between dilemma for meat, fish and dairy products was calculated using unweighted Kappa [32]. Sociodemographic, economic and lifestyle characteristics as well as food intake were compared across dilemma (yes, no) for meat, fish and dairy products. Continuous variables were presented as means ± SDs, and categorical variables as percentages. Multivariable logistic regression analyses were used to assess the associations between sociodemographic and economic characteristics and the composite variable of dilemma (yes, no) for meat, fish and dairy products (dependent variable). Analysis of covariance was performed to estimate the association between the composite variable of dilemma for meat, fish, dairy products and intake of these foods as well as adherence with food group guidelines (dependent variables). Logistic regression analysis was performed to estimate the association between dilemma for meat, fish and dairy products and mPNNS-GS score (dependent variable). Analysis of variance and logistic regression analyses were adjusted for, sex, age, education level, income, household composition, smoking status, physical activity, BMI and energy intake. Sensitivity analyses were performed on the implicit and explicit approaches separately. All tests of significance were two-sided, and a *P* value <0.05 was considered significant. All statistical analyses were performed using SAS software (version 9.3, SAS Institute Inc.).

## Results

### Characteristics of the sample

From the initial 122,091 subjects who received the questionnaire measuring food choice motives regarding sustainable foods, a total of 46,958 (38.5%) completed it. Among them, 402 subjects (0.9%) were excluded due to missing data for BMI, educational level or household composition. An additional 18,056 participants (38.5%) who had less than three dietary records available were excluded and 5565 participants (11.9%) due to underreporting, leaving 22,935 subjects (48.8% of those who completed the questionnaire) available for analysis (17,247 women and 5688 men).

Compared with excluded individuals, included individuals were more often men, were older, had a higher income and were less likely to have children (all *P* < 0.0001). They also showed a higher educational level (*P* < 0.01).

Percentage of participants who had a dilemma when choosing meat, fish or dairy products are shown in Table 1. More participants showed dilemma in the case of meat and fish than in the case of dairy products. In addition, the unweighted kappa coefficients between dilemmas for meat, fish and dairy products were as follow: meat vs fish (kappa = 0.43 (95% CI: 0.41–0.45), fish vs dairy products (0.33 (0.32–0.36)), meat vs dairy products (0.27 (0.26–0.29). Among participants, 22.4% presented a dilemma for at least one of these foodstuffs and 2.0% presented a dilemma for all these foodstuffs.

**Table 1:** Percentage of participants who presented a dilemma towards the choice of meat, fish and dairy products (*N* = 22,935, NutriNet-Santé study, 2013)

	Meat		Fish		Dairy products	
	Strongly agree/agree (%)	Dilemma (%)	Strongly agree/agree (%)	Dilemma (%)	Strongly agree/agree (%)	Dilemma (%)
Implicit approach						
I purchase [meat/fish/dairy products] for health issues	91.60	20.77^a^	86.24	19.55^a^	75.14	8.32^a^
I avoid purchasing [meat/fish/dairy products] for environmental issues	25.60		24.42		11.69	
Explicit approach						
I am torn between purchasing [meat/fish/dairy products] to follow dietary guidelines or limit purchase for environmental issues		31.94		31.75		14.79
Composite variable^b^		12.01		13.09		4.64

^a^Percentage of participants who answered “strongly agree/agree” to the two questions “I purchase [meat/fish/dairy products] for health issues” and “I avoid purchasing [meat/fish/dairy products] for environmental issues”

^b^Percentage of participants who presented a dilemma in both implicit and explicit approaches

Table 2 shows socio-demographic, economic, lifestyle and food intake characteristics of participants who demonstrated dilemma vs no dilemma for each of these foods. Table 3 shows results of the multivariable logistic regression analysis evaluating the association between sociodemographic and economic characteristics and dilemma toward food choice. Men were less likely to report dilemma, while individuals belonging to low income class and aged >50 years were more likely to report dilemma. Dilemma also increased with age. In the case of dairy products, individuals aged >30 years (and in particular >50 years), with a low education level and a low income were more likely to report dilemma compared with those without dilemma. Sensitivity analyses were performed on the implicit and explicit approaches separately. Analyses using the implicit question showed additional associations in the case of meat: individuals with a greater educational level and household including only one adult were more likely to report a dilemma. No major differences were observed between the composite variables and the explicit approach.

**Table 2:** Individual characteristics of participants across dilemma towards meat, fish and dairy products choice (N = 22,935, NutriNet-Santé study, 2013)

	All sample	Meat			Fish			Dairy product		
	N = 22,935	No dilemma	Dilemma	P^1^	No dilemma	Dilemma	P^1^	No dilemma	Dilemma	P^1^
		*N* = 18,138	*N* = 2767		*N* = 17,715	*N* = 2913		*N* = 20,221	*N* = 1001	
Sex, %				<0.0001			<0.0001			0.75
Women	75.2	76.6	81.1		76.4	83.3		77.5	77.0	
Men	24.8	23.4	18.9		23.6	16.7		22.5	23.0	
Age (years), %				<0.0001			<0.0001			<0.0001
18–30	9.0	9.1	6.7		8.6	6.5		9.4	2.5	
30–50	32.0	33.1	27.6		32.6	29.6		33.3	17.7	
50–65	40.6	40.5	44.2		40.9	45.1		40.0	54.8	
> 65	18.4	17.4	21.6		18.0	18.8		17.3	25.1	
Educational level, %				0.090			0.014			<0.0001
Primary	2.4	2.4	2.7		2.3	2.9		2.3	4.6	
Secondary	31.5	31.2	32.8		31.3	32.5		30.4	46.8	
University (≤ 3 years)	30.7	31.1	30.0		30.8	31.8		31.2	26.0	
University (> 3 years)	35.4	35.3	34.5		35.6	32.8		36.2	22.6	
Income (€/UC)^2^, %				0.27			0.0057			<0.0001
200	11.1	10.8	10.7		10.4	11.4		10.8	13.6	
1200–1800	22.8	22.7	23.3		22.6	24.1		22.8	24.1	
1800–2700	28.7	28.7	29.8		28.8	29.8		28.8	32.1	
700	33.2	33.6	32.3		34.1	30.7		33.6	25.0	
Missing data	4.3	4.2	3.9		4.2	4.0		4.1	5.3	
Household composition, %				0.0022			0.49			<0.0001
One adult	18.0	17.6	19.7		17.9	18.1		18.2	20.7	
Two adults	46.3	45.8	47.2		46.0	47.0		45.3	52.6	
Household with ≥1 child	13.8	14.5	12.8		14.2	14.0		14.6	7.1	
Household with ≥1 teenager	17.1	17.1	16.3		17.0	16.7		17.0	16.1	
Household with ≥1 child and 1 teenager	4.8	5.0	4.1		4.9	4.2		4.9	3.6	
Smoking status, %				0.0023			0.043			0.0011
Never smoker	49.9	49.6	52.6		49.9	51.3		50.4	49.4	
Former smoker	39.9	39.8	38.6		39.8	39.9		39.2	43.3	
Current smoker	10.2	10.6	8.8		10.3	8.8		10.5	7.3	
Physical activity, %				<0.0001			<0.0001			<0.0001
Low	18.4	19.3	14.6		18.5	16.3		18.7	14.1	
Moderate	37.8	37.9	39.4		38.1	38.3		38.5	32.8	
High	32.6	31.7	35.4		32.1	35.8		31.7	42.5	
Missing data	11.1	11.1	10.6		11.3	9.7		11.1	10.7	
BMI (kg/m^2^), %				<0.0001			<0.0001			0.032
< 25	69.6	68.6	75.7		69.1	74.6		70.0	66.3	
25–30	22.7	23.2	19.3		23.1	18.8		22.3	25.7	
> 30	7.70	8.20	5.1		7.90	6.60		7.7	7.8	
Dietary characteristics										
mPNNS-GS^3,4^	7.7 ± 1.6	7.6 ± 1.6	8.0 ± 1.6	<0.0001	7.7 ± 1.6	7.8 ± 1.6	<0.0001	7.6 ± 1.6	8.1 ± 1.6	<0.0001
Energy intake (kcal/day)	1893.8 ± 470.0	1894.1 ± 465.0	1850.8 ± 461.30	<0.0001	1891.3 ± 465.1	1848.5 ± 457.4	<0.0001	1891.1 ± 465.0	1827.5 ± 453.5	0.0002
Meat										
Intake (g/day)	69.6 ± 51.7	72.6 ± 51.8	60.3 ± 46.5	<0.0001	71.6 ± 51.8	60.5 ± 47.7	<0.0001	69.9 ± 51.6	65.2 ± 47.6	0.002
Adherence to dietary guidelines^5^	51.8	53.2	48.8	<0.0001	53.4	48.7	<0.0001	52.0	51.7	0.834
Fish										
Intake (g/day)	42.3 ± 44.5	43.8 ± 45.1	41.6 ± 42.3	0.0221	42.7 ± 44.7	42.9 ± 42.9	0.8066	42.3 ± 44.5	42.0 ± 42.6	0.85
Adherence to dietary guidelines^6^	56.0	55.4	65.5	<0.0001	57.8	58.3	0.60	55.6	62.6	<0.0001
Dairy products										
Intake (g/day)	191.3 ± 151.1	193.7 ± 150.5	178.0 ± 146.3	<0.0001	194.1 ± 151.1	175.4 ± 142.6	<0.0001	193.8 ± 150.7	181.1 ± 144.8	0.02
Adherence to dietary guidelines^7^	28.8	29.1	28.1	0.28	29.2	27.4	0.039	29.1	31.5	0.10

^1^P–values are based on chi-square tests

^2^CU: Consumption Units. One CU is attributed for the first adult in the household, 0.5 for other 569 persons aged 14 or older and 0.3 for children under 14

^3^Analyses are performed on a subsample of individuals (*N* = 17,685) for whom mPNNS-GS score could be assessed

^4^mPNNS-GS has a range of 0–13.5 points, with a higher score indicating a better overall diet quality

^5^Since there are no dietary guidelines available for meat, guidelines for meat/fish/eggs are considered in these analyses. Adherence to dietary guidelines for meat/fish/eggs corresponds to an intake of 1–2 servings/day

^6^Adherence to dietary guidelines for fish corresponds to an intake of fish ≥2 servings/week

^7^Adherence to dietary guidelines for dairy products corresponds to an intake between 2.5–3.5 servings/day (individuals <55 years) and between 2.5–4.5 servings/day (individuals ≥55 years)

**Table 3:** Multivariable logistic regression analyses showing odds ratios (OR) for risk of having a dilemma toward choice of meat, fish and dairy products across subgroups of individual (*N* = 22,935, Nutrinet-Santé study, 2013)^a^ ^b^

	Meat		Fish		Dairy products	
	OR (95% CI)	*P*	OR (95% CI)	*P*	OR (95% CI)	*P*
Sex		*< 0.0001*		*< 0.0001*		0.25
Women	1		1		1	
Men	0.73 (0.66–0.82)	*< 0.0001*	0.64 (0.58–0.71)	*< 0.0001*	0.91 (0.78–1.07)	0.25
Age (years)		*< 0.0001*		*< 0.0001*		*<0.0001*
18–30	1		1		1	
30–50	1.26 (1.05–1.45)	*0.011*	1.30 (1.09–1.56)	0.099	2.15 (1.39–3.32)	*0.0005*
50–65	1.78 (1.49–2.11)	*< 0.0001*	1.70 (1.43–2.02)	*< 0.0001*	4.90 (3.23–7.43)	*<0.0001*
> 65	2.12 (1.75–2.56)	*<0.0001*	1.73 (1.43–2.10)	*< 0.0001*	4.89 (3.17–7.53)	*<0.0001*
Educational level		0.37		0.62		*<0.0001*
University (> 3 years)	1		1		1	
University (≤ 3 years)	0.91 (0.82–1.01)	0.088	1.02 (0.92–1.13)	0.79	1.08 (0.89–1.30)	0.44
Secondary	0.93 (0.82–1.04)	0.20	0.98 (0.88–1.09)	0.21	1.56 (1.30–1.86)	*<0.0001*
Primary	0.94 (0.72–1.22)	0.63	1.13 (0.88–1.46)	0.31	1.73 (1.22–2.45)	*0.0022*
Income (€/UC)		*0.0063*		*<0.0001*		*<0.0001*
> 2700	1		1		1	
1800–2700	1.13 (1.01–1.26)	*0.028*	1.18 (1.06–1.32)	*0.0020*	1.46 (1.22–1.74)	*<0.0001*
1200–1800	1.19 (1.05–1.34)	*0.0046*	1.26 (1.12–1.42)	*<0.0001*	1.47 (1.20–1.79)	*0.0001*
< 1200	1.28 (1.10–1.50)	*0.0019*	1.39 (1.19–1.62)	*<0.0001*	2.10 (1.65–2.68)	*<0.0001*
Missing data	0.97 (0.78–1.21)	0.79	1.05 (0.85–1.30)	0.65	1.68 (1.23–2.29)	*0.0012*
Household composition		0.39		0.39		0.67
One adult	1		1		1	
Two adults	0.94 (0.83–1.05)	0.26	1.08 (0.96–1.20)	0.31	0.98 (0.83–1.17)	0.85
Household with ≥1 child	0.99 (0.84–1.17)	0.93	1.16 (0.99–1.37)	*0.028*	0.88 (0.64–1.20)	0.41
Household with ≥1 teenager	0.90 (0.78–1.04)	0.14	1.00 (0.87–1.15)	0.50	0.86 (0.69–1.08)	0.19
Household with ≥1 child and 1 teenager	0.85 (0.78–1.07)	0.16	0.93 (0.75–1.17)	0.22	0.89 (0.60–1.31)	0.56

^a^Multivariable logistic regression analyses were performed using “no dilemma” as reference

^b^All analyses are adjusted for all presented variables and additionally smoking status, physical activity and BMI

Table 4 shows results of differences in food intake, adherence to dietary guidelines and the mPNNS-GS between individuals who reported dilemma and those who did not. Participants who reported a dilemma toward meat, fish and dairy products purchase consumed less of these foods overall and had greater mPNNS-GS scores compared with those without dilemma. In the case of fish, differences were small, although significant. In addition, participants who presented a dilemma toward meat showed a better adherence to meat/fish/eggs guidelines. Additional analyses focusing on the explicit and implicit approaches separately showed similar results. Observed differences concerned intake of fish which did not differ significantly using the implicit approach (*P* > 0.05), and adherence to food group guidelines for meat/fish/eggs which was not significantly different in individuals with and without dilemma (both implicit and explicit) for meat (P > 0.05).

**Table 4:** Covariance analysis and multivariable logistic regression analysis showing the association between dilemma toward choice of meat, fish and dairy products and nutritional intake, adherence to food group guidelines and dietary quality (mPNNS-GS) (*N* = 22,935, Nutrinet-Santé study, 2013)^1^

		Intake (g/day)		Adherence to food group guidelines		Dietary quality (mPNNS-GS)^2,3^	
		Mean ± SD	*P* ^4,5^	OR (95% CI)	*P* ^6,7^	Mean ± SD	*P* ^4,8,9^
Meat	No dilemma	72.6 ± 51.8	*<0.0001*	1^6^	*0.0002*	7.6 ± 1.6	*<0.0001*
	Dilemma	60.3 ± 46.5		0.83 (0.76–0.92)		8.0 ± 1.6	
Fish	No dilemma	43.8 ± 45.1	*<0.0001*	1^10^	0.48	7.7 ± 1.6	*0.0048*
	Dilemma	41.6 ± 42.3		0.97 (0.88–1.07)		7.8 ± 1.6	
Dairy products	No dilemma	193.8 ± 150.7	*0.045*	1^11^	0.41	7.6 ± 1.6	*<0.0001*
	Dilemma	181.1 ± 144.8		1.07 (0.91–1.27)		8.1 ± 1.6	

^1^All analyses are adjusted for, sex, age, education level, income, smoking status, physical activity, BMI and energy intake

^2^Analyses are performed on a subsample of individuals (*N* = 17,685) for whom mPNNS-GS score could be assessed

^3^mPNNS-GS has a range of 0–13.5 points, with a higher score indicating a better overall diet quality

^4^P values are based on ANCOVA analyses

^5^Explained variance (r^2^) in the models were 0.073 for meat, 0.033 for fish and 0.024 for dairy products

^6^P values are based on multivariable logistic regression analyses

^7^Explained variance (r^2^) in the models were 0.010 for meat, 0.055 for fish and 0.027 for dairy products

^8^Since there are no dietary guidelines available for meat, guidelines for meat/fish/eggs are considered in these analyses. Adherence to dietary guidelines for meat/fish/eggs corresponds to an intake of 1–2 servings/day

^9^Explained variance (r^2^) in the models were 0.14 for meat, 0.13 for fish and 0.13 for dairy products

^10^Adherence to dietary guidelines for fish corresponds to an intake of fish ≥2 servings/week

^11^Adherence to dietary guidelines for dairy products corresponds to an intake between 2.5–3.5 servings/day (individuals <55 years) and between 2.5–4.5 servings/day (individuals ≥55 years)

## Discussion

Around one-tenth of the individuals participating in this study showed dilemma between health and environmental motives when purchasing meat and fish, while dilemmas were less frequent in the case of dairy products. Participants who reported dilemma for meat and fish purchasing were more likely to be older, women and to have a low income, while in the case of dairy products; they were more likely to be older, with a low education level and income. Participants reporting dilemmas for meat and dairy products consumed less of the corresponding foods and had a better dietary quality (mPNNS-GS score). In addition, individuals with dilemma towards meat had a lower adherence to meat/fish/eggs guidelines.

### Dilemma occurrence when purchasing meat, fish and dairy products

Our study showed that a significant number of individuals were torn between health and environmental considerations when purchasing meat or fish (around 10%), while it was the case for 5% only in the case of dairy products. In addition, around one-fifth of the participants had a dilemma for at least one of these foods. To our knowledge, this study is the first to investigate potential dilemmas between health and environmental considerations when purchasing animal food. Occurrence of dilemmas is expected to be greater when there is a contradiction between health and environmental guidelines, but also when a product has a strong perceived environmental impact. In agreement, meat and in particular the one from ruminants present the highest greenhouse gas emissions together with fish, while dairy products score lower [33]. However, it has been demonstrated that it is possible to create a diet that is healthy and sustainable [16, 34, 35] without performing drastic dietary changes [16, 36] or increasing the cost to the consumer [16]. The public is aware of a number of dietary guidelines [6] which are widely disseminated [5], while awareness of what constitutes a low environmental impact diet is likely to be lower given the lack of information on this topic. In addition, consumer difficulty to understand what constitutes a good, balanced diet from a health, environmental, ethical or other perspective has been acknowledged [18] and can lead to dilemmas. More and more authors suggest that dietary guidelines should integrate recommendations for environmental sustainability [17, 37] as it has been done in other countries such as Germany [19]. It is likely that the level of dilemma will be lower in these countries.

### Socio-demographic and economic characteristics associated with dilemma

In our study, women declared more dilemmas in the case of meat and fish compared with men, while no difference was observed for dairy products. In a previous study, women were also more likely to agree that they were ‘not sure whether to buy farmed fish’ [20]. Women are known to be more interested in healthy eating [38–41] and to be more often concerned by environmental issues [9, 42–44]. In particular, they were more willing to reduce their meat consumption [45]. However, women have been shown to rate almost all eating motivation as more frequently influencing eating behavior than men [38, 46], which may be due to women being more preoccupied with food in general [9, 47]. This could also partly explain why they declare more dilemma in general.

Our data showed that older subjects were more likely to perceive dilemma compared with younger subjects, for all food groups considered. Older age groups are known to be more interested in healthy eating compared with younger subjects [20, 40, 41, 46, 48]. It is however still unclear whether environmental concerns vary with age, with studies showing more pro-environmental attitudes in younger individuals [49, 50], while older subjects have been shown to be more familiar with the “meatless meals” topic [45] and to have greater ethical concern [9, 20, 48]. The greater health concerns observed in older subjects might partly explain why dilemma also increases with age. It has also been suggested that older consumers may have had a bias to give higher ratings on scales generally [48]. In our study, the risk of dilemma occurrence increased linearly with age in the case of meat and fish while dairy products showed a threshold effect starting at 50 years, with particularly high odds ratio. French dietary guidelines advise an increase of dairy products to four portions per day from the age of 55 years due to the high prevalence of osteoporosis in the elderly population [51]. This may increase dilemmas in older subjects also concerned by the environment.

Level of education had an influence on dilemma occurrence in the case of dairy products only, with less educated individuals showing more dilemmas. In a previous study, level of education had no influence on health motives to select food [38, 39]. In addition, attitude towards fish was not modified by a composite variable of socio-economic characteristics (educational level, occupation, household income and individual food expenditure) [20]. However, better educated individuals have been shown to express more pro-environmental attitudes compared with less educated individuals [49, 50]. People with higher education levels have more nutrition knowledge [52, 53] and might therefore be more able to compile nutritional and environmental information.

Finally, our data showed that individuals with low income had more dilemma compared with higher income individuals. The literature showed no influence of income on health motives underlying the selection of food [38] or intention to purchase sustainably sourced food [54]. Only minor effects for income was observed when it came to purchasing [43] or consuming [55] organic food. It is possible that individuals with low income perceive more dilemma in general when purchasing foods, since they have to take into account cost barriers, and as a consequence also perceived more health vs environmental dilemmas. In the case of meat, symbolic role of this food, such as its contribution to physical strength and energy, and social norms in low socioeconomic categories could also weight on the decision to maintain its important status in meals in this socioeconomic group [56], and may lead to dilemma related to the knowledge of the high environmental impact of this food.

### Association between dilemma and nutritional intake

Individuals with dilemma for meat and dairy products showed lower intake of these foods. These results suggest that dilemmas are translated into actual change in behavior and that barriers preventing changes can, at least partially, be overcome [15]. Our results showed limited association in the case of fish. In the literature, participants were less likely to report purchase of fish from sustainable source if they were confused about which type of fish they should be eating to protect fish stocks [20]. Dilemma for fish might potentially impact the type of fish purchased rather than the quantity consumed. Individuals reporting dilemmas for meat and dairy products had a diet of better quality overall. Our data therefore suggest that having environmental concerns is not inconsistent with a diet of adequate nutritional quality as previously shown [16, 34, 35]. In the literature, other studies showed that individuals with higher level of interest in health [57–60], or environment [57] had healthier food choice behavior.

The major strength of our study is its large sample size providing high statistical power. Also, this study includes a vast heterogeneous sample of volunteers in whom a wide range of socio-demographic and lifestyle characteristics were assessed so as to effectively control for potential confounding factors [61]. Further strength of the current study was the use of repeated 24 h records to assess nutritional intake that were validated against urinary biomarkers [24, 25]. Another strength is the use of a composite variable of dilemma including both implicit and explicit approaches. In the case of meat, an explicit measure of dilemmas led to 30% of individuals with dilemmas whereas an implicit led to 20% of individuals with dilemmas only. These results highlight the importance to use an instrument with various items to measure the adhesion to a theory [62]. The choice of a composite variable might also have influenced the results. However, additional analyses were performed on implicit and explicit approaches separately and showed very similar results. The main limitation of the study was its cross-sectional design, preventing inference of causality. Caution is also needed when generalizing our results, since the NutriNet-Santé study is a long-term cohort focusing on nutrition and participants are recruited on a voluntary basis, implying that they might have increased health consciousness and interest in nutritional issues as well as healthier lifestyle. Individuals with dilemma may also have a healthier profile overall, which was partially accounted for in the models by adjusting on smoking, physical activity, BMI and energy covariables. Finally, the sample size can also be a constraint since it produces significant results even though differences are small, but it enables highly accurate estimates.

## Conclusion

A number of individual showed dilemmas between health and environmental motives when purchasing meat and dairy products. Subjects at risk to present a dilemma were older, women and had lower income. Participants reporting dilemmas for meat and dairy products consumed less of the corresponding food but showed a better dietary quality. These data therefore suggest that having environmental concerns is not contradictory with adherence to nutritional guidelines. Public health strategies aiming at encouraging healthy and environmentally friendly food choices need to better understand consumers’ motives when purchasing food. National dietary guidelines could be adapted to take into account both health and environmental issues as it has already been done in some countries. However, these results must be confirmed by studies assessing dilemmas in real-life settings. Further work exploring dilemmas across populations from diverse backgrounds and with different level of concern toward the environment is needed to refine our knowledge when setting up effective nutrition policies.
